# Analytical typology of multiprofessional primary care models

**DOI:** 10.1186/s12875-018-0731-8

**Published:** 2018-04-05

**Authors:** Damien Contandriopoulos, Mélanie Perroux, Aurore Cockenpot, Arnaud Duhoux, Emmanuelle Jean

**Affiliations:** 1 School of Nursing, University of Victoria, PO Box 1700, STN CSC, Victoria, British-Columbia V8W 2Y2 Canada; 2 Faculty of Nursing, Université de Montréal, C.P. 6128 succ. Centre-ville, Montréal, Québec H3C 3J7 Canada; 3 School of Nursing, Université du Québec à Rimouski, 300, allée des Ursulines, C. P. 3300, succ. A, Rimouski, Québec G5L 3A1 Canada

**Keywords:** Primary health care, Comprehensive health care, Nursing care, Health services research

## Abstract

**Background:**

There is only limited evidence to support care redefinition and role optimization processes needed for scaling up of a stronger primary care capacity.

**Methods:**

Data collection was based on a keyword search in MEDLINE, EMBASE and CINAHL databases. Three thousand, two hundred and twenty-nine documents were identified, 1851 met our inclusion criteria, 71 were retained for full-text assessment and 52 included in the final selection. The analysis process was done in four steps. In the end, the elements that were identified as particularly central to the process of transforming primary care provision were used as the basis of two typologies.

**Results:**

The first typology is based on two structural dimensions that characterize promising multiprofessional primary care teams. The first is the degree to which the division of tasks in the team was formalized. The second dimension is the centrality and autonomy of nurses in the care model. The second typology offers a refined definition of comprehensiveness of care and its relationship with the optimization of professional roles.

**Conclusions:**

The literature we analyzed suggests there are several plausible avenues for coherently articulating the relationships between patients, professionals, and care pathways. The expertise, preferences, and numbers of available human resources will determine the plausibility that a model will be a coherent response that is appropriate to the needs and environmental constraints (funding models, insurance, etc.). The typologies developed can help assess existing care models analytically or evaluatively and to propose, prospectively, some optimal operational parameters for primary care provision.

## Background

Demographic changes, technological developments, and fiscal constraints are jeopardising the sustainability of health systems. Technical innovations and rapid growth in the intensity of care being provided are exerting significant pressure on available resources [1, 2]. Likewise, data on demographic changes and healthcare expenditures suggest that continued reliance on current healthcare provision models to address population health needs is likely to put enormous pressure on public finances [3]. Evidence suggests that public health systems need to change significantly to preserve their capacity to maintain universal access to healthcare [4, 5]. Capacity strengthening in primary care provision is widely considered to be an approach with the potential to simultaneously reinforce health system sustainability and accessibility, continuity of care, and ultimately population health [2, 5–10].

In this study, primary care is defined according to the terminology of the Institute of Medicine, as “*the provision of integrated, accessible healthcare services by clinicians who are accountable for addressing a large majority of personal healthcare needs, developing a sustained partnership with patients, and practicing in the context of family and community*” [6]. It is interesting to note that this definition does not focus on the types of clinicians who are providing care. Rather, it emphasizes the nature of the response being provided to meet the needs.

To address the above-cited challenges, there is growing evidence not only that health systems need to evolve to reinforce the provision of primary care, but that, in fact, the nature of that care itself will need to change [11–16]. In particular, professional teams need to move toward more collaboration and a broadening of fields of practice [17–19]. Numerous convergent data suggest that one possible progression would be to expand the interdisciplinary composition of primary care teams, the scope of practice of non-physician team members, and their intersectoral action [20–26]. In particular, greater use of an extended scope of nursing practice is likely to enhance both accessibility of care and efficiency of service delivery [12, 27–32]. Likewise, the care on offer should evolve to encompass a wider basket of services, less intensive and more appropriate interventions as well as more interventions targeting the determinants of health [1, 2, 4, 5, 16, 33–40]. Such a shift toward increased prevention and interventions that are more intersectoral has a better chance of curbing the growing burden of chronic disease management [22, 41].

However, there is little data available on the operational characteristics of care provision models that would be most promising to move in that direction. Generally speaking, the available literature indicates there are several plausible avenues to improve service provision performance, and that their relative merits are contingent on several clinical, organizational, and systemic factors. Against this backdrop, we conducted a realist review of the literature, grounded in a logic analysis approach, with the aim of producing conceptual tools to support the transformation of primary care models. Inductive analysis of the selected publications led to the development of two typologies. These typologies allow us to assess existing care models analytically or evaluatively and to propose, prospectively, some optimal operational parameters for primary care provision.

## Methods

### Logic analysis and realist review

To better understand the characteristics of high-performing multiprofessional primary care teams, we adopted a methodology based on two complementary approaches: Pawson and colleagues’ work on the realist review approach [42–45], and the work of Brousselle and colleagues on logic analysis, and more specifically, on reverse logic analysis [46].

The logic analysis approach was developed in the context of evaluating complex interventions. The underlying principle is to interpret the available scientific data with a view to understanding the causal processes that explain the outcomes of the intervention under study. Reverse logic analysis, on the other hand, is a prospective approach in which scientific data are used to identify the conditions and operational mechanisms of an intervention that would make it possible to achieve pre-determined objectives.

The principle underlying realist review is conceptually very similar. It is based on the idea that systematic literature review methods developed for studying the effectiveness of clinical interventions are not appropriate for analyzing complex interventions [43]. In the latter case, a “generative” approach [45] is needed that incorporates existing knowledge, either to propose new plausible interventions or to adjust existing interventions. This type of knowledge synthesis generates theoretical propositions regarding the intervention’s functioning.

The method used here, inspired by these two sources, draws from reverse logic analysis the notion of a relatively systematic synthesis of available knowledge and then incorporating this knowledge into efforts aimed at pre-determined objectives. From a realist review, we derive the notion of an approach that is more generative than summative, in which the data are interpreted inductively to propose recommendations for practice.

This work is part of a larger research project and will serve as a conceptual framework for analyzing practice in the pilot sites studied in this project [47].

### Analytical model

The objective of the present study was to better understand the structural characteristics and operational processes of high-performing multiprofessional primary care teams. We used primarily Donabedian’s conceptual framework [48] to categorize the information gleaned from the articles analyzed and to identify the causal links among: 1) the structures of primary care provision models, 2) the processes of care provision and management, and 3) the outcomes produced.

### Definition of performance

To normatively assess the desirability of care provision models, we used a definition of performance inspired by the work of Shortell and colleagues [49]. This definition conceives of performance as combining four components: 1) accessibility; 2) quality of care; 3) efficiency; and 4) collective learning capacities [47]. Accessibility is defined, based on Donabedian [48, 50], as the fit between structures of production, on the one hand, and society’s needs and their geographic distribution, on the other.

The definition of quality used here is based on an adaptation of the work done by the teams of Pineault, Beaulieu, and Haggerty [51–54] on operationalizing the measurement of primary care quality. Quality of care is defined as the combination of technical quality, continuity, and comprehensiveness. Technical quality is made up of three components: the quality of the act itself, the appropriateness of care, and the quality of communication. Continuity is defined, based on the work of Haggerty et al. [53], as the fact that a patient is treated by the same professional or the same team over time (relational continuity) and that services are harmoniously integrated with each other (management continuity). We interpret harmonious integration of care as occurring on two levels. On the one hand, care and services should be organized in such a way that medical care, nursing care, social services, curative practices, and preventive practices are harmoniously linked, sustained over time, and optimal (horizontal integration), and, on the other hand, that there are linkages between the different service levels (vertical integration) [54–56]. Finally, comprehensiveness refers to a care structure’s ability to respond in an integrated manner to all of a patient’s needs. Comprehensiveness encompasses two dimensions that make up the scope of patient management: taking into account all of a patient’s needs (whole person focus) and providing a complete basket of services (scope of services) [53].

Efficiency is defined here, based on A Brousselle, J Lachaine and AP Contandriopoulos [57], as the ratio between quality of care and resource use. This definition corresponds to technical efficiency, which is aimed at minimizing costs for any given outcome.

Finally, to implement organizational, administrative, and clinical practices that will foster the efficient production of good-quality services adapted to patients’ preferences, teams and organizations must have a strong learning capacity. Learning capacity here refers to the organization’s collective capacity to adapt and to assimilate new learning and new practices [49]. This requires a collective commitment to identifying and resolving problems [58, 59].

### Documentary search strategy

In a first step, we conducted a keyword search on MEDLINE that was subsequently adapted to the EMBASE and CINAHL databases (see endnote^1^). To be considered eligible, articles had to have been published in either English or French in peer-reviewed journals after 1999 and be focused on an intervention carried out in an Organisation for Economic Co-operation and Development (OECD) country. That search identified 3204 references, of which 1826 met our inclusion criteria.

In addition to the results of the keyword search in clinical databases we included articles known to the researchers and results from online search engines. Some documents cited in the bibliographies of the articles retained were also added. This approach was inspired by the work of Greenhalgh and colleagues [60, 61]. These deliberative approaches identified an additional 25 articles.

**Fig. 1:**
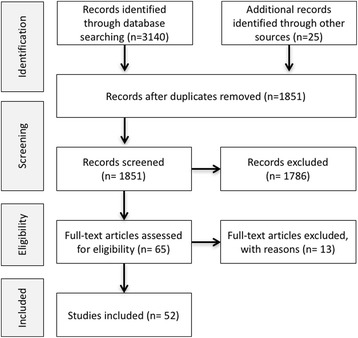
PRISMA 2009 Flow Diagram

The second step was to sort the articles based on the criterion of relevance to our objective of better understanding the structural characteristics and operational processes of high-performing multiprofessional primary care teams. This initial sorting was based on titles and abstracts. We retained only articles documenting primary care provision by multiprofessional teams in which nurses played a significant role. Articles dealing with services provided in hospital or that did not pertain to a general primary care clientele were excluded (e.g. emergency services, ambulatory care, rehabilitation). At the end of this step, 65 of the 1826 articles identified by keywords and 6 of the 25 articles from other sources were retained for full-text analysis. Three of the authors (DC, AC, and MP) subsequently read the full text of the selected articles and discussed the relevance of each article. In the end, 52 articles were retained for analysis. Figure 1 provides a flow diagram of the process [62].

### Analysis process

The analysis process was done in four steps. First, the articles retained for analysis were positioned according to their main contribution(s) according to a structure–process–outcomes grid. In a second step, numerous subcategories were inductively identified based on elements discussed in the articles. For example, many articles discussed the process of redefining professional roles [63], which was identified as a sub-category of processes. Likewise, the literature we examined included several analyses of the effects of primary care provision structures on certain clinical outcomes [41, 64, 65]. The work of categorizing and inductively identifying subcategories was conducted in parallel by each of the three readers. The classification exercise primarily served as a basis for discussing the presumed or tested causal links between the categories and sub-categories identified.

In a third step, the three readers organized a group discussion on the subcategory tree structure and the presumed causal links. The starting point for discussion was a series of iteratively produced causal models in the form of diagrams linking the subcategories among themselves. As soon as a satisfactory causal model was stabilized, the discussion turned toward identifying the conceptual tools needed to envision how existing primary care models might be modified to become higher-performing models, as defined above.

From this step, the group discussion then focused on elements that stood out as being particularly central to the process of transforming the primary care service offer. The elements that were found to be essential were specifically the links of accountability between care teams and the patient populations under their responsibility, the continuity and comprehensiveness of service provision, the operational efficiency of the model, the care pathways involved in the teams’ different methods of patient management, team composition, and especially the process of redefining professional roles and the scope of practice of non-physician professionals.

Finally, in a fourth step, the elements that were identified as particularly central to the process of transforming primary care provision were compared among themselves, which led progressively to the development of the typologies presented in the following section. As such, the typologies are not a synthesis of the causal models identified, but rather are “generative” models, in the sense of Pawson [45], that are useful for reflecting on the plausible characteristics of high-performing primary care models.

## Results and discussion

### Care team structure

The literature analysis identified two structural dimensions to characterize multiprofessional primary care teams. The first was the degree to which the division of tasks in the team was formalized. At one extreme were formal approaches based on explicit procedures that specified which service would be provided by which professional, to which patient, and at what point in time. At the other extreme were organic approaches based on mutually adjustable mechanisms, where professionals adapted to structural circumstances and to patients’ characteristics in deciding on care processes.

**Fig. 2:**
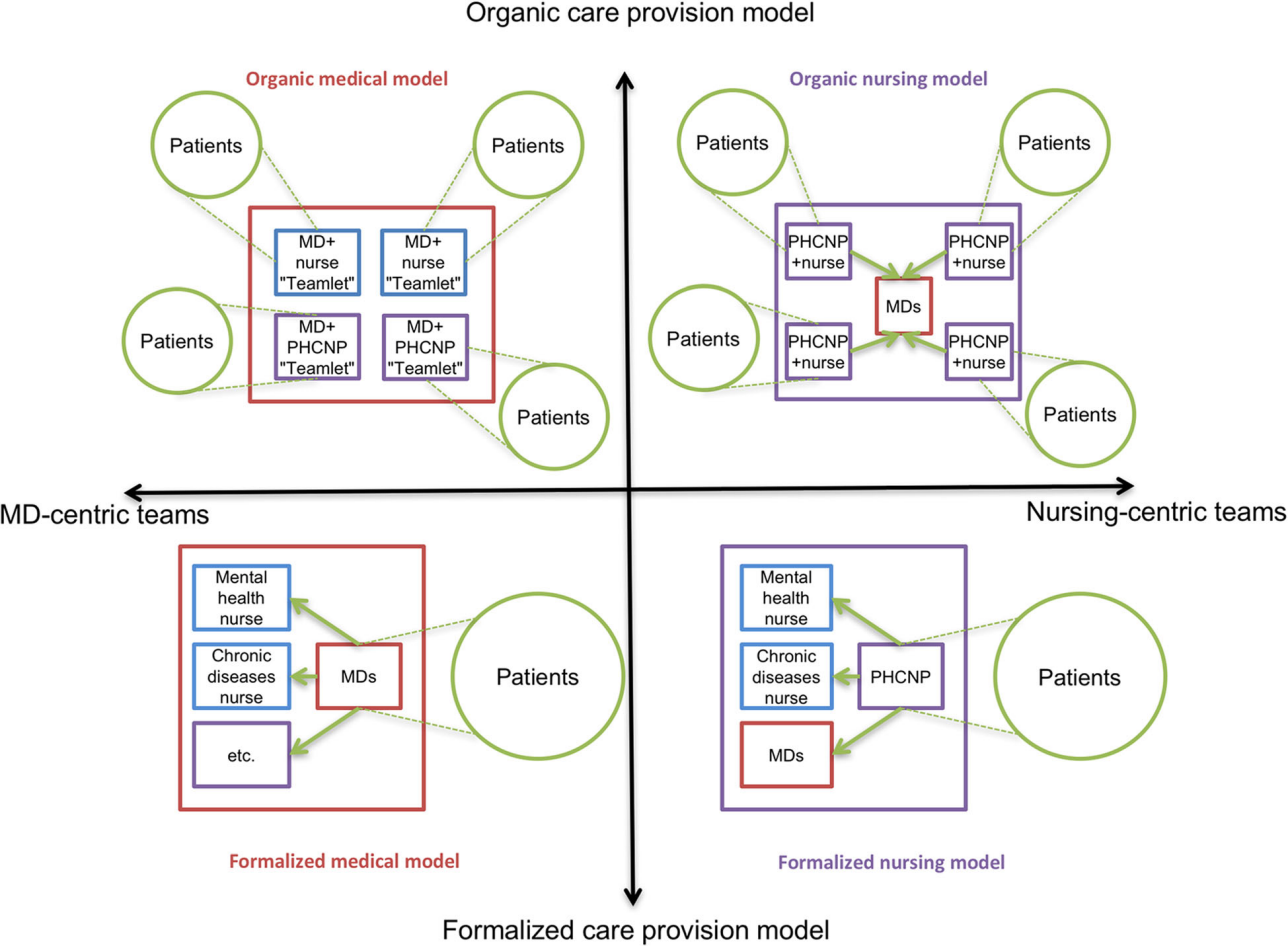
Two dimensional typology of primary care models

The second dimension was the centrality and autonomy of nurses—regardless of their exact title or level of training—in the care model. Some models were based primarily on nurses as the front-line professionals, while others were based mainly on physicians. In the articles analyzed, we did not find any primary care models based on professionals other than physicians or nurses. The two structuring dimensions can be used to construct a matrix divided into quadrants and to devise four ideal types of primary care models (see Fig. 2).

#### Formalized model with medical hub

The formalized model with a medical hub is probably the dominant model in OECD countries [66]. In this model, a group of physicians jointly manages a panel of patients. Here the core professional is a physician, who establishes the care plan in the initial consultation and determines the diagnosis and treatment plan. Subsequently, depending on the patient’s needs, the physician may refer the patient for further follow-up in what we here call “modules” of non-medical treatment (e.g. psychological, metabolic disease, or nutritional problems). These modules, centred around a specialized or disciplinary practice, include technical acts and patient education, and offer relatively autonomous follow-up by non-physician professionals of certain common problems or problems specific to chronic diseases. When the professionals in charge of service provision in these modules are nurses, they generally work in areas of specialization (e.g. diabetes, asthma, mental health, pregnancy care). For example, once a physician has diagnosed and created a treatment plan for a diabetic patient, that patient can be regularly followed by a nurse specialized in that disease. Often, that nurse will be autonomously responsible for adjusting the patient’s medication and assessing the progress of the disease. In most cases, there are a certain number of patient management protocols stipulating the point at which the patient should be seen again by the physician, such as an annual physical exam or every second visit. When the patient’s health status changes, he or she is referred back to the physician.

#### Formalized model with nursing hub

The formalized model with nursing hub differs from the preceding model in that the core professional is a nurse, generally a primary healthcare nurse practitioner (PHCNP). This form represents one of the possible operationalizations of a *nurse-led clinic*, such as exists in Ontario or in the United States [67–69]. We consider this model formalized because, as in the medical model described above, the PHCNP determines the diagnosis and establishes the treatment plan and, for some of their needs, refers patients to the same types of care modules described above. However, the particular feature of this model is that one of the PHCNP’s options is to refer the patient to a physician. For instance, the PHCNP looks after patients’ needs that fall within the PHCNP’s field of practice and set of skills. Patients whose needs are more complex or who require a treatment or diagnostic test that requires a physician’s intervention are referred to physicians.

We will return to these points, but in both these formalized models, the main challenge is to ensure the best possible flow for patients among the different resources within the team, especially for patients with multimorbidities. This is a horizontal integration challenge.

#### Organic model with medical hub

In comparison with the two preceding models that both have formal procedures for referring patients within the team, organic models are based on the comprehensive management of all needs by small multidisciplinary teams. What characterizes organic models is that service provision is based on micro-teams—an approach much publicized by the Veteran’s Health Administration (VHA) system in the U.S. under the name “teamlets” [70]. In a medical-hub model, for example, physician–nurse duos, or physician–NP–nurse trios, or other combinations of two to four professionals work closely together in a joint patient management model [28, 71]. This means patients being followed by a micro-team will see one, two or all of the professionals in the team at each visit, based on flexible parameters. In terms of comprehensiveness of care, each micro-team can handle a wide range of primary care needs. Patient pathways are less fragmented than in the two preceding models. Roles are less formally defined in micro-teams, and professionals decide who will see which patient for what service and at what time by mutual accommodation according to the circumstances. The patient panels for each team might be constituted randomly depending on team availability, but in a same group, there can also be micro-teams working in specific areas (e.g. perinatal care, pediatrics, geriatrics, etc.).

#### Organic model with nursing hub

Lastly, the organic model with a nursing hub, the most hypothetical of the four models, is also based on micro-teams; here, however, the centre of gravity is a PHNCP working jointly with other professionals. This is another possible form of *nurse-led clinic.* When a patient’s needs require medical expertise, nursing micro-teams will call on one or more physicians, who either belong to the clinic or who are outside the clinic and with whom there are partnership arrangements. The level of collaboration between the PHCNP and the nurses depends on which acts are legally reserved, and practice overlaps are self-regulated in each micro-team. What distinguishes this model from the formalized nursing-hub model is the integrated and joint management of a panel of patients by a micro-team of professionals whose centre of gravity is a PHCNP.

Theoretically, the main challenge of organic models is to optimize the process by which every professional in each micro-team is being used to their fullest capacity. The risks are that subsidiarity in task definition might not be pushed as far as it could go and that the range of services might be limited, especially in the organic nursing-hub model.

### Care comprehensiveness and role structuring

The second conceptual tool developed from our literature review have to do with the comprehensiveness of care offered, how work and patient management are organized within the team, and the collaboration processes upon which this management is based. In practice, the scope of services actually offered in a primary care vary significantly. In many care models comprehensiveness of care is suboptimal in relation to what is suggested in the literature [54]. The “one problem per visit” model of some walk-in clinics is an extreme example of this situation, as is the practice of refusing to manage certain clienteles (more serious mental health problems, dual diagnoses involving mental health and addiction, etc.).

Referring back to the dimensions set out in the performance model presented above, we recall that comprehensiveness of care consists, on the one hand, of responding to the full set of patients’ needs (*whole person focus*) and, on the other, of offering a complete basket of services (*scope of services*). Our analysis of the literature has led us to refine this definition.

First, the consideration of all needs must be conceptualized at the population level rather than at the individual level. A care team that only looks after young patients without any complex needs could easily meet all their needs but would not, in our view, be providing comprehensive care. Here we conceptualize the notion of responding to all needs in relation to the needs of the population to whom care is being provided and with no exclusion criteria being applied.

Second, we conceptualize the offer of a complete basket of services in terms of the complexity of care provided. By defining primary care as a response to “a large majority of personal healthcare needs” [6] we are in fact relying on a definition of comprehensive primary care. There are, implicitly, more specialized services that require expertise, a technical platform, or specific resources that fall within the purview of specialized or hospital medicine. In this respect, offering a complete basket of services also means ensuring that primary care teams manage the full complexity of common care delivery and do not refer their patients unnecessarily or too readily to specialized services.

**Fig. 3:**
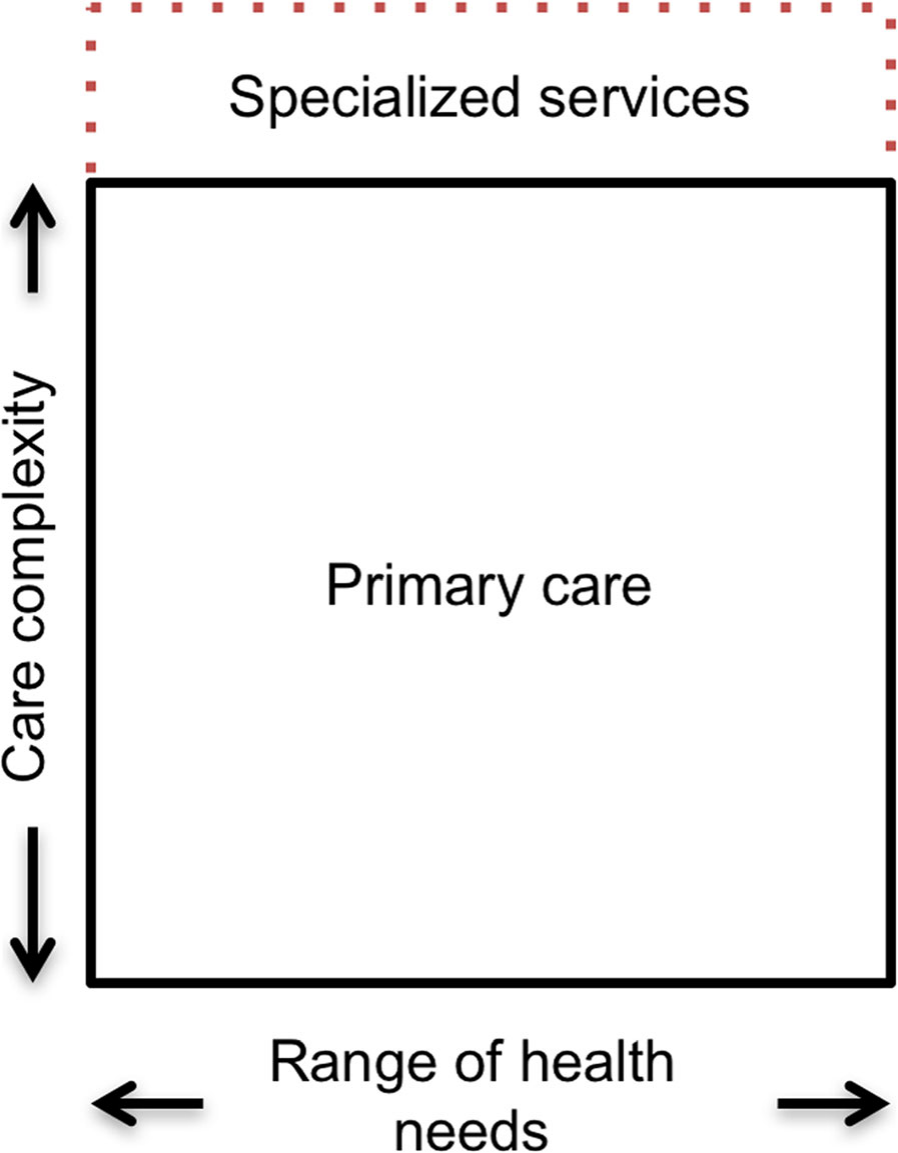
Conceptualization of care comprehensiveness and professional roles

Given the two dimensions of comprehensiveness we have defined, this concept can be visualized as a square in which the horizontal sides represent all population needs, while the vertical sides represent complexity of care (Fig. 3).

#### Applying the typology to primary care comprehensiveness

**Fig. 4:**
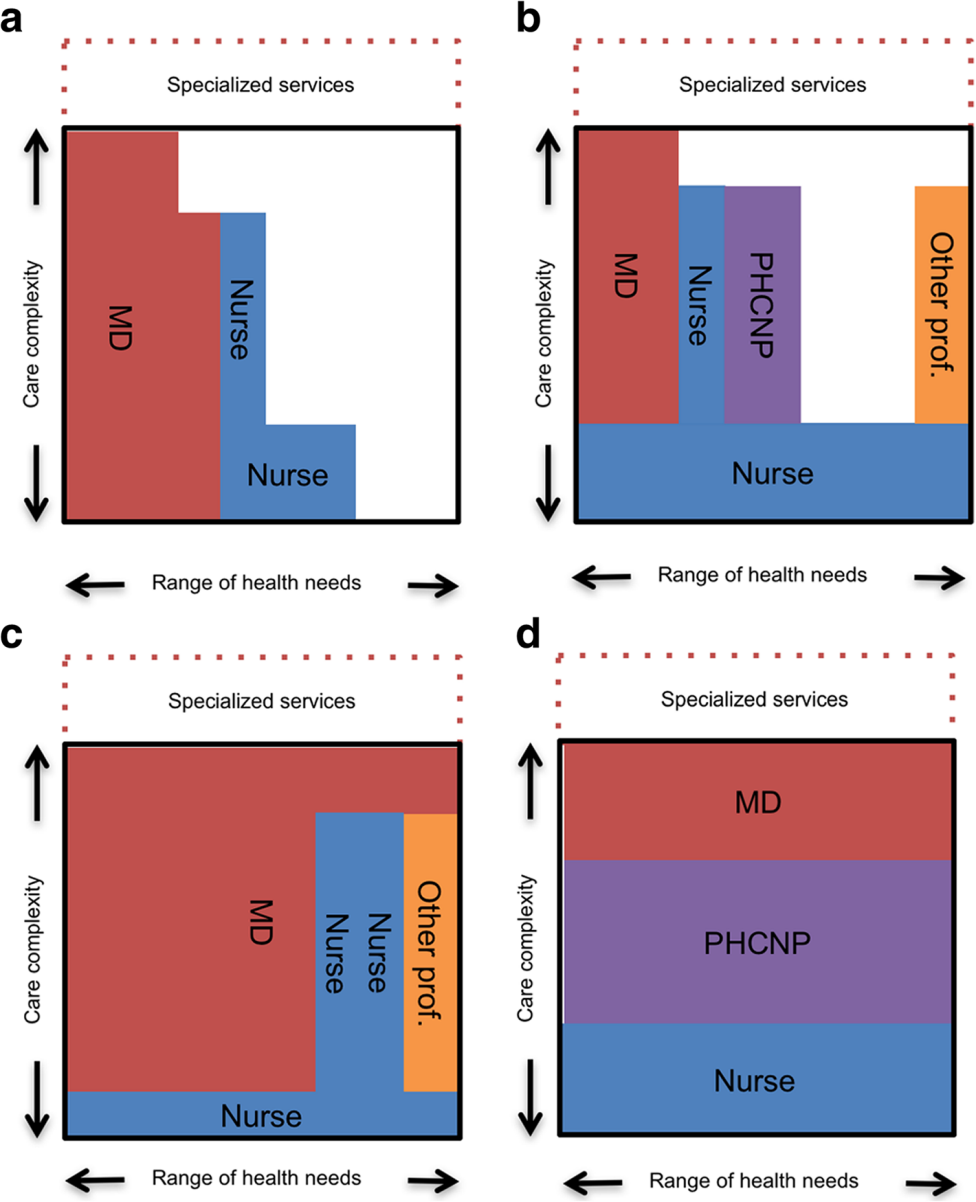
Illustration of four hypothetical primary care models: **a** and **b** are examples of models with limited scope while **c** and **d** are examples of models providing a full scope of services

In our view, this comprehensiveness typology is useful on two levels. Firstly, it can be used to visually represent the actual comprehensiveness of care provided by a team as compared to a normative ideal of the optimal comprehensiveness. By comparing the services actually provided by a team against what is done in other teams or what the scientific literature identifies as best practices, it is possible to characterize certain models as offering a limited comprehensiveness. Thus, in Fig. 4 models A and B some zones in the optimal primary care square are empty, whereas ideally they should all be filled.

At a second level, this typology can be used to conceptualize the role of each professional or of each professional group in the provision of comprehensive care. We use the term role to describe the services a health professional actually performs owing to the available resources, skills, knowledge, patients needs and preferences and so forth [72]. The typology makes it possible not only to visualize the relative contributions of each professional group to the services provided, but to discern how the sharing of roles and responsibilities is structured in relation to the two dimensions of range of needs and complexity of care. As a tool for transforming practice, the aim of this typology is to maximize potential comprehensiveness by linking role definition with available professional resources.

In Fig. 4, the two hypothetical models presented, A and B, do not cover all needs (limited scope). Model A, in the top left quadrant, for example, is a primarily medical model with limited scope and limited complexity. In such a model, it quickly becomes impossible for patients to be followed for complex problems and for health problems not handled by this team. Model B, in the upper right quadrant, is a second model, this time multiprofessional, of limited comprehensiveness. In this hypothetical team, there is a response to the full scope of needs, but for several types of care and services, that response is only for problems of low complexity. For example, a patient with a routine mental health problem such as anxiety would be seen by a nurse who, rather than setting up a regular follow-up within the team, would probably refer the patient to psychological resources in another care structure.

Conversely, the two hypothetical models C and D cover the entire scope of primary care. Model C, in the lower left quadrant, is primarily medical, while Model D, in the lower right quadrant, is primarily a nursing model. In both cases, these models incorporate the idea—based on the principle of subsidiarity—that low-complexity care is assigned to nurses. It would be possible, however, to imagine models that also incorporate this principle using other professionals for certain services. Models C and D differ, though, in how their role definitions are structured. Model C is based on tasks being shared according to what we described earlier as non-physician patient management modules. It is possible to imagine that all patients are seen initially by a nurse, who performs a first needs assessment, after which patients are followed and treated by a physician. However, still in this model, for certain visits or certain patients who come for a specific type of follow-up, the care could be provided by non-physician professionals. In our view, it is plausible to hypothesize that the models described as formalized in the structural typology presented in the preceding section would be represented by a more vertical division of roles when visualized using the present model.

In contrast, Model D is based on tasks being shared according to complexity. All patients, whatever their needs or their characteristics, are followed by a nurse for the simple aspects, and by a PHCNP for most other needs. The physician, in the role of primary care “specialist”, sees only those patients with complex needs. The representation is simplified, but it is conceivable that, depending on the nature of the care and needs, the horizontal cut-offs would vary according to types of needs. We believe it is plausible to hypothesize that the models in which professionals are divided horizontally are more consistent with a structure considered organic in the typology presented in the preceding section, with less formalized boundaries and greater recourse to clinical judgment and mutual accommodation to manage overlaps.

Obviously such a representation remains very schematic and could never adequately convey all the sophistication of actual service provision by primary care teams. Nevertheless, we believe this conceptualization is interesting in that it incorporates the concept of role definition as a parameter of comprehensiveness optimization. As observed in the introduction, high-performing primary care models will move toward broader coverage of needs and a redrawing of professional boundaries. In operationalizing the decisions needed to make these transitions successfully, it is thus important that both these processes be considered together.

### Expansion and extension of roles

As stated in the introduction, one of the main challenges facing most health systems today is how to enlarge the contribution of primary care in the total healthcare services offer. Based on the two dimensions of the care comprehensiveness typology, this would involve, on the one hand, increasing the range of population needs for which primary care teams would be responsible and the level of complexity of care provided in primary care. In the diagram we have proposed (see Fig. 2), this mean drawing a large square, i.e., the normative definition of what constitutes primary care.

On the other hand, it is also important to ensure that most teams develop collaboration practices and roles that enable them, in practice, to actually cover all those needs. On our proposed diagram, this would mean filling all the space in the square, i.e., aligning the operationalization of professional roles with the response to needs.

However, while our proposed care comprehensiveness typology is useful for thinking about the different performance components of primary care models in an integrated way (such as accessibility and quality) it does not take efficiency into account. For example, a primary care team relying exclusively on physicians that would cover the full range of needs and all complexity of care (a full red square) would be perfectly adequate in terms of comprehensiveness, but would probably, in most countries, be characterized by an excessively high per-patient cost.

Analysis of the literature suggests, not surprisingly, that expanding the primary care services offer while maintaining or improving efficiency calls for greater use of multiprofessional teams. In reflecting on the growth of nursing practice, Richards et al. [73] recommend differentiating between *expanding* and *extending* practice. Building on their idea, we address this issue from a broader interprofessionnal perspective.

*Expanding* the practice of non-physician professionals essentially means retaining current professional roles but increasing the number of non-physicians in the teams to increase the intensity of treatment or quantity of services provided. Expanding non-physician practice—in particular, nursing—can improve physician productivity or team productivity as a whole.

*Extending* practice, on the other hand, means that non-physician professionals develop new areas of practice that would allow them, for example, to manage certain clienteles or certain visits more autonomously, based on a substitution rationale [29]. Such practice extension can take the form of increasing the complexity of care provided by non-physician professionals (vertical extension in our diagram), or of broadening the range of needs covered (horizontal extension in our diagram). In all cases, what characterizes the notion of extension is that non-physician professionals’ roles transcend established professional boundaries.

From a logical standpoint, some combination of expansion and extension of practice would likely be needed to increase the space allocated to primary care in the overall healthcare services offer while improving health system efficiency. The data we analyzed, however, offer no indication of what proportions of the two avenues constitute the optimal response. According to the data available in the literature, the choice is made idiosyncratically, primarily based on the professional and financial resources available. The real impact on efficiency of combining the expansion and extension of non-physician professionals’ practice will depend, in each case, on how consistently roles are defined and operationalized in practice [74].

## Conclusions

Referring back to the original objective of the present literature review, which was to understand the structural characteristics and operational processes of high-performing multiprofessional primary care teams, our analytical review of the literature leads us to four conclusions.

First, developing a coherent response to the challenges facing health systems today will require a rethinking of both the structural aspects of accountability for patient management (who is responsible for following which patients, and for what care?) and the redefinition of professional roles that this entails. As indicated by our proposed typology of structural characteristics of primary care models, there are several plausible avenues for articulating the relationships between patients, professionals, and care pathways. We found no solid evidence upon which to determine whether one or another of the quadrants of our typology is more promising in terms of performance. Our interpretation is that there is probably a contingency relationship between these structural parameters [75] and performance. In other words, for a primary care team’s operational performance, what counts is that the model uses available resources coherently. In particular, the expertise, preferences, and level of available human resources will determine the plausibility that a model will be a coherent response that is appropriate to the needs and environmental constraints (funding models, insurance, etc.).

Second, even though it is heuristically interesting to differentiate among the various dimensions of primary care performance, in reflecting on their operationalization it is clear that they are strongly interrelated. For example, according to the model developed here, improving accessibility involves ensuring that primary care models cover the full range of health needs. When care models cover only a portion of needs (limited scope and/or complexity), this means in practice that certain health problems or clienteles are being under-served. There is therefore a direct logical link between this situation and problems of accessibility and care continuity. When specific populations are excluded (such as homeless persons, patients with a dual diagnosis of mental health disorder and addiction, or immigrants—three cases of populations often under-served by the dominant care models), the problem is felt at the intersection of comprehensiveness and accessibility. For specific health problems (such as HIV, hepatitis C, mental health disorders, or when rapid access is required for semi-urgent acute problems), patients often have to contend with being treated in silos, in which different teams, in different healthcare structures, each manage just one part of the problem. In this case, poor comprehensiveness presents significant challenges for care continuity. In this respect, our comprehensiveness typology can be useful for rethinking several different dimensions of primary care performance in a more integrated manner.

Third the literature we analyzed included many analyses of the challenges involved in transforming care structures and practice models [63, 76–80]. Managing change processes and redefining roles require skills and resources that are not always available on the ground. In the performance model we have used, these elements are included in the notion of learning capacity. Learning capacity is both a result of performance and, at the same time, an essential condition for achieving positive results in terms of accessibility, efficiency, and quality [49]. Learning capacity is required for teams to be able to better manage complexity [81] and adapt to the frequent changes occurring in today’s health systems [82–84]. Some authors posit that learning capacity is very closely linked with success or failure in the implementation of innovations [85], and even with organizational survival. As such, our research team is continuing to work on identifying how collective learning capacity is manifested in the transformation of care provision models.

In closing, as mentioned earlier, the literature we reviewed does not allow us to form any simple or reliable judgment on the structural characteristics or operational processes of primary care teams. Our anticipation of this outcome explains the choice of approach used here. In effect, the type of literature review conducted here makes it possible, up to a certain point, to bypass this limitation by connecting available knowledge in a generative way. The proposed typologies are generative, in that they allow us to consider how several structuring performance parameters of primary care models might fit together. Ultimately, what matters is that care models are developed in which resources, structures, professional roles, care pathways, and population needs come together in a coherent whole. We believe the models proposed in this article are useful tools to this end.

## Abbreviations

OECD: Organisation for Economic Co-operation and Development
PHCNP: Primary healthcare nurse practitioner
VHA: Veteran’s Health Administration

## Footnotes

1 An optimized search strategy was developed for each database. As an example, the CINHAL one could be summarized as: [primary health care OR ambulatory care OR family practice OR community health services OR group practice OR patient centred medical home OR walk-in centre] **AND** [nurse led OR nurse-managed centres OR advanced nursing practice OR registered nurses OR nurse practitioners OR nursing role] **AND** [quality of health care OR health service accessibility OR organizational efficiency OR multidisciplinary care team OR interprofessional relations OR model of care OR delivery model].

## References

[1] Commission on Social Determinants of Health (2007). Achieving health equity: from root causes to fair outcomes. Interim statement.

[2] Rittenhouse DR, Shortell SM, Fisher ES (2009). Primary care and accountable care -- two essential elements of delivery-system reform. N Engl J Med.

[3] Clavet N-J, Duclos J-Y, Fortin B, Marchand S, Michaud P-C (2013). Les dépenses en santé du gouvernement du Québec, 2013-2030 : projections et déterminants.

[4] Ham C, Dixon A, Brooke B (2012). Transforming the delivery of health and social care: the case for fundamental change.

[5] Starfield B, Yushi L, Macinko J (2005). Contribution of primary care to health systems and health. Milbank Q.

[6] Donaldson MS, Yordy KD, Lohr KN, Vanselow NA (1996). Primary care: America's health in a new era (committee on the future of primary care, Institute of Medicine).

[7] Feachem RG, Sekhri NK, White KL, Dixon J, Berwick DM, Enthoven AC (2002). Getting more for their dollar: a comparison of the NHS with California's Kaiser Permanente. BMJ.

[8] Haggerty J, Pineault R, Beaulieu M-D, Brunelle Y, Gauthier J, Goulet F, Rodrigue J (2008). Practice features associated with patient-reported accessibility, continuity, and coordination of primary health care. Ann Fam Med.

[9] Hutchison B (2013). From hall to now: reflections on Canadian Medicare from a primary care perspective. Emmett hall memorial lecture.

[10] Macinko J, Starfield B, Shi L (2003). The contribution of primary care systems to health outcomes within Organization for Economic Cooperation and Development (OECD) countries, 1970–1998. Health Serv Res.

[11] Christensen CM, Grossman JH, Hwang J (2009). The innovator's prescription: a disruptive solution for health care.

[12] Laurant MG, Hermens RP, Braspenning JC, Akkermans RP, Sibbald B, Grol RP (2008). An overview of patients’ preference for, and satisfaction with, care provided by general practitioners and nurse practitioners. J Clin Nurs.

[13] Naylor M, Kurtzman ET (2010). The role of nurse practitioners in reinventing primary care. Health Aff.

[14] Sibbald B, Laurant M, Scott A, Saltman RB, Rico A, Boerma W (2006). Changing task profiles. Primary care in the driver's seat? Organizational reform in European primary care.

[15] Commission on the Reform of Ontario's Public Services (2012). Public services for Ontarians: a path to sustainability and excellence. Chapter 5: health.

[16] Marmot M (2007). Achieving health equity: from root causes to fair outcomes. Lancet.

[17] Bodenheimer TS, Smith MD (2013). Primary care: proposed solutions to the physician shortage without training more physicians. Health Aff.

[18] Lavis JN, Boyko JA (2009). Evidence brief: strengthening primary health care in Canada.

[19] Delamaire M-L, Lafortune G (2010). Nurses in advanced roles: a description and evaluation of experiences in 12 developed countries. OECD health working papers no 54.

[20] Bailey P, Jones L, Way D (2006). Family physician/nurse practitioner: stories of collaboration. J Adv Nurs.

[21] Bamford D, Griffin M (2008). A case study into operational team-working within a UK hospital. Int J Oper Prod Man.

[22] Litaker D, Mion L, Kippes C, Mehta N, Froliks J (2003). Physician-nurse practitioner teams in chronic disease management: the impact on costs, clinical effectiveness, and patients’ perception of care. J Interprof Care.

[23] Hasselback P, Saunders D, Dastmalchian A, Alibhai A, Boudreau R, Chreim S (2003). The Taber integrated primary care project: turning vision into reality.

[24] Bonsall K, Cheater FM (2008). What is the impact of advanced primary care nursing roles on patients, nurses and their colleagues? A literature review. Int J Nurs Stud.

[25] Baker GR, MacIntosh-Murray A, Porcellato C, Dionne L, Stelmacovich K, Karen B (2008). High performing healthcare systems: delivering quality by design.

[26] Doherty RB, Crowley RA (2013). Health and public Committee of the American College of physicians. Principles supporting dynamic clinical care teams: an American College of Physicians position paper. Ann Int Med.

[27] Reay T, Patterson EM, Halma L, Steed WN (2006). Introducing a nurse practitioner: experiences in a rural Alberta family practice clinic. Can J Rural Med.

[28] DiCenso A, Matthews S (2007). Report of the nurse practitioner integration task team submitted to the Ontario minister of health and long-term care.

[29] Laurant MG, Reeves D, Hermens R, Braspenning J, Grol R, Sibbald B (2005). Substitution of doctors by nurses in primary care. Cochrane database Syst rev.

[30] Martin-Misener R, Downe-Wamboldt B, Cain E, Girouard M (2009). Cost effectiveness and outcomes of a nurse practitioner-paramedic-family physician model of care: the long and Brier Islands study. Prim Health Care Res Dev.

[31] Richardson G, Maynard A, Cullum N, Kindig D (1998). Skill mix changes: substitution or service development?. Health Policy.

[32] Thille P, Rowan MS (2008). The role of nurse practitioners in the delivery of primary health care: a literature review. Unpublished report submitted to Health Canada.

[33] Grady D, Redberg RF (2010). Less ss more: how less health care can result in better health. Arch Intern Med.

[34] Krogsbøll LT, Jørgensen KJ, Grønhøj Larsen C, Gøtzsche PC (2012). General health checks for reducing illness and mortality. Cochrane database systematic rev.

[35] Krogsbøll LT, Jørgensen KJ, Grønhøj Larsen C, Gøtzsche PC (2012). General health checks in adults for reducing morbidity and mortality from disease: Cochrane systematic review and meta-analysis. BMJ.

[36] Fisher ES, Bynum JP, Skinner JS (2009). Slowing the growth of health care costs — lessons from regional variation. N England J Med.

[37] Hadler NM (2008). Worried sick: a prescription for health in an overtreated America.

[38] Cassels A (2012). Seeking sickness: medical screening and the misguided hunt for disease.

[39] Moseley JB, O'Malley K, Petersen NJ, Menke TJ, Brody BA, Kuykendall DH, Hollingsworth JC, Ashton CM, Wray NP (2002). A controlled trial of arthroscopic surgery for osteoarthritis of the knee. N England J Med.

[40] Wright CJ, Chambers GK, Robens-Paradise Y (2002). Evaluation of indications for and outcomes of elective surgery. CMAJ.

[41] Griffiths P, Maben J, Murrells T (2011). Organisational quality, nurse staffing and the quality of chronic disease management in primary care: observational study using routinely collected data. Int J Nurs Stud.

[42] Pawson R, Tilley N (1997). Realistic evaluation.

[43] Pawson R, Greenhalgh T, Harvey G, Walshe K (2005). Realist review--a new method of systematic review designed for complex policy interventions. J Health Serv Res Policy.

[44] Pawson R (2006). Evidence-based policy: a realist perpective.

[45] Pawson R (2002). Evidence-based policy: the promise of `realist synthesis. Evaluation.

[46] Brousselle A, Champagne F (2011). Program theory evaluation: logic analysis. Eval Prog Plan.

[47] Contandriopoulos D, Duhoux A, Roy B, Amar M, Bonin J-P, Da Silva RB, Brault I, Dallaire C, Dubois C-A, Girard F (2015). Integrated primary care teams (IPCT) pilot project in Quebec: a protocol paper. BMJ Open.

[48] Donabedian A (1988). The quality of care. How can it be assessed?. JAMA.

[49] Shortell SM, Schmittdiel J, Wang MC, Li R, Gillies RR, Casalino LP, Bodenheimer T, Rundall TG (2005). An empirical assessment of high-performing medical groups: results from a national study. Med Care Res Rev.

[50] Wilson K, Rosenberg MW (2004). Accessibility and the Canadian health care system: squaring perceptions and realities. Health Policy.

[51] Beaulieu M-D, Haggerty JL, Beaulieu C, Bouharaoui F, Lévesque J-F, Pineault R, Burge F, Santor DA (2011). Interpersonal communication from the patient perspective: comparison of primary healthcare evaluation instruments. Healthc Policy.

[52] Haggerty JL, Burge F, Beaulieu M-D, Pineault R, Beaulieu C, Lévesque J-F, Santor DA, Gass D, Lawson B (2011). Validation of instruments to evaluate primary healthcare from the patient perspective: overview of the method. Healthc Policy.

[53] Haggerty JL, Burge F, Lévesque J-F, Gass D, Pineault R, Beaulieu M-D, Santor D (2007). Operational definitions of attributes of primary health care: consensus among Canadian experts. Ann Fam Med.

[54] Pineault R, Lévesque J-F, Roberge D, Hamel M, Couture A (2008). Primary care services organisational models and the population's care experience.

[55] Pineault R (2008). Accessibility and continuity of care: a study of primary healthcare in Québec. Research report presented to the Canadian Institutes of Health Research (CIHR) and the Canadian health services research Fondation (CFSRF).

[56] Waddington C, Egge D (2008). Integrated health services– what and why? Technical brief no.1, 2008.

[57] Brousselle A, Lachaine J, Contandriopoulos AP, Brousselle A, Champagne F, Contandriopoulos AP, Hartz Z (2009). L’évaluation économique. Concepts et méthodes d’évaluation des interventions.

[58] Decuyper S, Dochy F, Van den Bossche P (2010). Grasping the dynamic complexity of team learning: an integrative model for effective team learning in organisations. Educ Res Rev.

[59] Raes E, Boon A, Kyndt E, Dochy F (2015). Measuring team learning behaviours through observing verbal team interaction. J Workplace Learning.

[60] Greenhalgh T, Robert G, Macfarlane F, Bate P, Kyriakidou O (2004). Diffusion of innovations in service orgrnizations: systematic review and recommendations. Milbank Q.

[61] Greenhalgh T, Robert G, Macfarlane F, Bate P, Kyriakidou O, Peacock R (2005). Storylines of research in diffusion of innovation: a meta-narrative approach to systematic review. Soc Sci Med.

[62] Moher D, Liberati A, Tetzlaff J, Altman DG, The PRISMA Group (2009). Preferred reporting items for systematic reviews and meta-analyses: the PRISMA statement. PLoS Med.

[63] Rosen R, Mountford L (2002). Developing and supporting extended nursing roles: the challenges of NHS walk-in centres. J Adv Nurs.

[64] Iglesias B, Ramos F, Serrano B, Fàbregas M, Sánchez C, García MJ, Cebrian HM, Aragonés R, Casajuana J, Esgueva N (2013). A randomized controlled trial of nurses vs. doctors in the resolution of acute disease of low complexity in primary care. J Adv Nurs.

[65] Venning P, Durie A, Roland M, Roberts C, Leese B (2000). Randomised controlled trial comparing cost effectiveness of general practitioners and nurse practitioners in primary care. BMJ.

[66] Contandriopoulos D, Brousselle A, Breton M, Sansgter-Gormley E, Kilpatrick K, Dubois C-A, Brault I, Perroux M (2016). Nurse practitioners, canaries in the mine of primary care reform. Health Policy.

[67] Heale R, Butcher M (2010). Canada's first nurse practitioner-led clinic: a case study in healthcare innovation. Nurs Leadersh.

[68] Martínez-González NA, Djalali S, Tandjung R, Huber-Geismann F, Markun S, Wensing M, Rosemann T (2014). Substitution of physicians by nurses in primary care: a systematic review and meta-analysis. BMC Health Serv Res.

[69] Wong FKY, Chung LCY (2006). Establishing a definition for a nurse-led clinic: structure, process, and outcome. J Adv Nurs.

[70] Rosland A-M, Nelson K, Sun H, Dolan ED, Maynard C, Bryson C, Stark R, Shear JM, Kerr E, Fihn SD (2013). The patient-centred medical home in the veterans health administration. Am J Manag Care.

[71] Bush NJ, Watters T (2001). The emerging role of the oncology nurse practitioner: a collaborative model within the private practice setting. Oncol Nurs Forum.

[72] Nelson S, Turnbull J, Bainbridge L, Caulfield T, Hudon G, Kendel D, Mowat D, Nasmith L, Postl B, Shamian J (2014). Optimizing scopes of practice: new models of care for a new health care system. Report of the expert panel appointed by the Canadian academy of health sciences.

[73] Richards A, Carley J, Jenkins-Clarke S, Richards DA (2000). Skill mix between nurses and doctors working in primary care-delegation or allocation: a review of the literature. Int J Nurs Stud.

[74] Liu N, D’Aunno T (2012). The productivity and cost-efficiency of models for involving nurse practitioners in primary care: a perspective from queueing analysis. Health Serv Res.

[75] Donaldson L, Clegg SR, Hardy C, Nord WR (1996). The normal science of structural contingency theory. Handbook of organization studies.

[76] Ainsworth B, Hayward S (2010). Developing an innovative model of care for nurse-led walk-in centres in the ACT. Aust Nurs J.

[77] Heale R (2012). Overcoming barriers to practice: a nurse practitioner-led model. J Am Acad of Nurse Pract.

[78] Hoff T (2013). Medical home implementation: a sensemaking taxonomy of hard and soft best practices. Milbank Q.

[79] Krothe JS, Flynn B, Ray D, Goodwin S (2000). Community development through faculty practice in a rural nurse-managed clinic. Public Health Nurs.

[80] Tuepker A, Kansagara D, Skaperdas E, Nicolaidis C, Joos S, Alperin M, Hickam D (2014). “We've not gotten even close to what we want to do”: a qualitative study of early patient-centred medical home implementation. J Gen Intern Med.

[81] McGrath RG (1991). Exploratory learning, innovative capacity and managerial oversight. Acad of Manage J.

[82] Antonacopoulou EP, Sheaffer Z (2014). Learning in crisis rethinking the relationship between organizational learning and crisis management. J Manage Inquiry.

[83] Balasubramanian BA, Cohen DJ, Davis MM, Gunn R, Dickinson LM, Miller WL, Crabtree BF, Stange KC (2015). Learning evaluation: blending quality improvement and implementation research methods to study healthcare innovations. Implementation Sci.

[84] Engler ES, Jones SL, Van de Ven AH (2013). Organizing healthcare for changing markets: the case of Ascension health. J Organ Design.

[85] Jiménez-Jiménez D, Sanz-Valle R (2011). Innovation, organizational learning, and performance. J Bus Res.

